# Moxidectin induces Cytostatic Autophagic Cell Death of Glioma Cells through inhibiting the AKT/mTOR Signalling Pathway

**DOI:** 10.7150/jca.46697

**Published:** 2020-08-03

**Authors:** Jingjing Liu, Hongsheng Liang, Saadia Khilji, Haitao Li, Dandan Song, Chen Chen, Xiaoxing Wang, Yiwei Zhang, Ning Zhao, Xina Li, Aili Gao

**Affiliations:** 1 School of Life Science, Northeast Agricultural University, Harbin, Heilongjiang, China.; 2 Department of Neurosurgery, The First Affiliated Hospital of Harbin Medical University, Harbin, Heilongjiang, China.; 3 Department of Cellular and Molecular Medicine, Faculty of Medicine, University of Ottawa, Ottawa, Ontario, Canada.; 4 Department of Pharmacy, The First Affiliated Hospital of Harbin Medical University, Harbin, Heilongjiang, China.; 5 College of Life and Health Sciences, Northeastern University, Shenyang, Liaoning, China.

**Keywords:** Moxidectin, Glioma, Autophagy, AKT/mTOR

## Abstract

Moxidectin (MOX), a broad-spectrum antiparasitic drug, has been characterized as a potential anti-glioma agent. The main objective of this study was to explore autophagy induced by MOX in glioma U251 and C6 cells, and the deep underlying molecular mechanisms. In addition, the effects of autophagy on apoptosis in glioma cells were tested. Autophagy was measured by transmission electron microscopy (TEM), immunofluorescence, western blot and immunohistochemistry. Cell viability was detected with MTT and colony formation assay. The apoptosis rate was measured by flow cytometry and terminal deoxynucleotidyl transferase dUTP nick end labelling (TUNEL). Additonally, autophagy inhibition was achieved by using 3-Methyladenine (3-MA) and chloroquine (CQ). U251-derived xenografts were established for examination of MOX-induced autophagy on glioma *in vivo*. Firstly, our research found that MOX stimulated autophagy of glioma cells in a dose-dependent manner. Secondly, we found that MOX induced autophagy by inhibiting the AKT/mTOR signalling pathway. Thirdly, inhibition of autophagy could reduce apoptosis in MOX-treated glioma cells. Finally, MOX induced autophagy, and autophagy increased the apoptosis effect of MOX on U251 *in vivo*. In conclusion, our data provide evidence that MOX can induce autophagy in glioma cells, and autophagy could increase MOX-induced apoptosis through inhibiting the AKT/mTOR signalling pathway. These findings provided a new prospect for the application of MOX and a novel targeted therapy for the treatment of gliomas.

## Introduction

Glioma is one of the most common and malignant tumors of the central nervous system, characterized by well-adapted to poorly immunogenic and hypoxic conditions 1, 2. The current therapies mainly consist of tumour excision, adjuvant radiotherapy and chemotherapeutics. Despite the diverse strategies that have been proposed to improve the current situation, the prognosis for patients with glioma cancer still remains poor 3, 4. It is necessary to develop new anticancer drugs and assess the molecular mechanisms of glioma cell death.

Recent studies have indicated that some macrocyclic lactones (MLs) were effective against proliferation of tumor cells 5, 6. Kobayashi T et al. investigated the potential role of clarithromycin addition to lenalidomide and dexamethasone therapy (BiRd) in multiple myeloma 7. Previous publications have demonstrated that ivermectin induced cytostatic autophagy by blocking the PAK1/Akt axis in Breast Cancer 8. Meanwhile, Ivermectin inhibited angiogenesis, growth and survival of glioblastoma 9. These findings provided insights into the anticancer efficacy of MLs, which support a preclinical rational to explore broadening the evaluation of MLs for the treatment of tumors. Among these MLs, moxidectin (MOX) belongs to the milbemycin family and is third generation macrocyclic lactone with potent endectocide activity, and is similar in structure of the avermectins 10, 11. Specifically, MOX is a new oral treatment for human scabies which provides a solid foundation for considering its potential translation to human diseases 12. Our previous study demonstrated that MOX had an effect on inhibiting viability of glioma cells *in vitro* and *in vivo* by inducing apoptosis and cell cycle arrest. Thus, we propose that MOX might be a potent and promising agent to combat glioma 13.

Conventional anticancer therapies primarily triggered apoptosis to promote cancer cell death 14. However, accumulating evidence suggested that apoptosis and autophagic cell death could coexist in different chemotherapy drugs to induce cancer cell death 15, 16. According to morphological appearance, cell death could be classified into apoptosis, autophagy, or necrosis 17. Moreover, tumors are more autophagy-dependent than normal tissues, suggesting that there is a therapeutic window 18. Autophagy is a self-degrading process of cellular components characterized by double-membrane autophagosomes which sequester excess or defective organelles and fuse with lysosomes or vacuoles for breakdown by resident hydrolases 19-21. The role of autophagy in cancer is complex, and this complexity is illustrated by autophagy promoting or suppressing tumorigenesis 22-24. Therefore, inhibiting or forcing autophagic machinery would be useful in drug cancer treatment 25. These studies suggest autophagy lysosomal pathway is one such target that may be a promising avenue for the development of novel therapeutic strategies for treating gliomas 26.

In this study, we first detected MOX-induced autophagy in U251 and C6 cells, followed by a discussion of the the deep molecular mechanisms of autophagy. Furthermore, we explored the relationship between apoptosis and autophagy induced by MOX *in vitro* and* vivo.*

## Materials and Methods

### Cell lines and cell culture

Cell lines human U251 glioma and rat C6 glioma were obtained from The First Affiliated Hospital of Harbin Medical University (Harbin, China). Cells were routinely cultured in a humidified incubator at 37°C under 5% CO_2_ atmosphere, in Dulbecco's minimal essential medium (DMEM; HyClone, USA) supplemented with fetal bovine serum (FBS), 100 U/mL penicillin and 0.1 mg/mL streptomycin.

### Reagents and chemicals

MOX was purchased from Sigma-Aldrich/Merck KGaA (Darmstadt, Germany; European Pharmacopoeia Reference Standard), and a purity of > 95%. MOX was dissolved in dimethyl sulfoxide (DMSO). FBS was provided by Zhejiang Dayhang biological technology (Hangzhou, China). Penicillin-streptomycin Solution, Annexin V-FITC Apoptosis Detection Kit was purchased from Beyotime Biotechnology (Shanghai, China). MTT, diaminobenzidine (DAB), polyvinylidene fluoride (PVDF) membranes and hematoxylin were purchased from Sigma-Aldrich/Merck KGaA. The reagents 3-methyladenine (3-MA), chloroquine (CQ) and DMSO were obtained from Sigma-Aldrich/Merck KGaA. Antibodies used in this study were against LC3B (CST, 2775S, USA), P62 (CST, 5114T, USA), AKT (CST, 9272S, USA), Phospho-AKT (Ser473) (Beyotime, AA329, China), mTOR (CST, 2972S, USA), Phospho-mTOR (S2448) (Boster, BM4840, China), p70S6K (Bioss, bs-6370R, China), Phospho-p70S6K (Ser417) (Bioss, bs-5668R, China), GSK3β (Bioss, bs-0023RR, China), Phospho-GSK3β (Bioss, bs-0028RR, China), Ki67 (Bioss, bs-23103, China), β-actin (Sigma-Aldrich, A1978, Germany), goat anti-rabbit immunoglobulin G (IgG) (H+L)-horseradish peroxidase (HRP; SunGene GmbH, LK2001, Germany) and goat anti-mouse IgG (H+L)-HRP (OriGene Technologies, ZB-2305, USA).

### Transmission electron microscopy

Transmission electron microscopy (TEM) was used to analyze morphological characteristics of autophagy. U251 and C6 cells were seeded in incubation bottles for 24 h. Then cells were treated with MOX for 48 h, the untreated cells served as control group. Then the cells were harvested, washed and fixed overnight with 2.5% glutaraldehyde containing 1% tannic acid at 4°C. After washing, the cells were dehydrated using a graded series of ethanol solutions, 10 min at a time, transferred to propylene oxide and embedded in epon araldite (Polysciences, Inc., Polybed 812, USA). The ultrathin sections were observed using a JEM-100CX transmission electron microscope (Shanghai Yongming Automatic Equipments Co. Ltd., H-7650, China) and representative images were photographed and analyzed.

### Plasmid transfection

MOX treatment resulted in marked autophagy induction in glioma cells, which was proved by GFP-LC3 puncta. Transfection with GFP-LC3 plasmid was obtained from The First Affiliated Hospital of Harbin Medical University. U251 and C6 cells (1×10^6^ cells/well) were seeded in 6-well plates overnight at 37°C. Lipofectamine 2000 (Invitrogen, USA) and GFP-LC3 plasmid were added according to the manufacturer's instructions for 4 h. Then, cells were fixed with 4% paraformaldehyde (Sigma) for 30 min, washed three times with phosphate-buffered saline (PBS). The treated cells were visualized with an inverted fluorescence microscope (Feica, Germany). The cells treated with DMSO were used as control. Nuclei were stained with DAPI (Invitrogen, USA).

### Western blot assay

Western blot was used to investigate the expression of autophagy-associated proteins. After treatment, U251 and C6 cells were lysed with RIPA buffer supplemented with PMSF (Beyotime, China) and quantified using the BCA protein concentration assay kit (Beyotime, China). Protein samples were separated by sodium dodecyl sulfate-polyacrylamide Gel electrophoresis (Bio-Rad, USA) and electrotransferred to a PVDF (0.22 μm). After blocking with 5% non-fat milk, the membranes were incubated with primary antibodies overnight at 4°C. The next day, the membranes were labelled with secondary antibody. Signals were detected using MiniChemi Imager (SageCreation, Beijing, China). β-actin was used as the endogenous control.

### Cell viability assay

Cell viability was examined by 3-(4,5-dimethylthiazol-2-yl)-2,5-diphenyltetrazolium bromide (MTT) assay. C6 (2×10^4^ cells/well) and U251 (1×10^4^ cells/well) cells were seeded into a 96-well plate respectively overnight in 5% CO_2_ at 37℃. And the cells were pretreated with CQ (15 μM) for 1 h before being exposed to MOX. Next, Cells were incubated with 5 mg/mL MTT reagent for another 4 h at 37°C. After the medium was carefully removed, 150 μL of DMSO was added and agitated to dissolve the formazan crystals. The absorbance was recorded at 490 nm on an enzyme-linked immunosorbent assay reader (HUADONG-Y, China).

The long-term effects of MOX on tumor cell proliferation were analyzed with a colony formation assay. U251 and C6 cells were seeded into a 6-well plate. Next the cells were treated with CQ (15 μM) for 1 h before being exposed to MOX and cultured in medium for 13 days. The medium was refreshed every three days. Thereafter, the cells were washed with PBS three times, fixed with 4% paraformaldehyde and stained with 0.1% crystal violet (Sigma, USA). Quantification of colony formation was also performed using ImageJ software.

### Flow cytometry

The cells were stained with Live-Dead to discriminate living cells. C6 and U251 cells (2.5×10^5^ cells/well) were seeded into 6-well plates and pretreated with CQ (15 μM) for 1 h before being exposed to MOX. Cells were harvested, washed in ice-cold PBS, and then resuspended in PI/Annexin-V solution for apoptosis analysis according to the manufacturer's instructions. Apoptosis ratio was measured using a BD Biosciences FACSCalibur flow cytometer (BD Biosciences, Franklin Lakes, NJ, USA). The results were quantified using the Cell Quest software (BD Biosciences, USA), and apoptosis was calculated as percentage of early and late apoptotic cells.

### Tumor xenograft mouse models

All animal experiments were carried out in Harbin Vic Biological Technology Development Co., Ltd., Harbin, China (Experiment number: SY-2017-Mi-027). 5-week-old female Balb/c nude mice (Beijing vitonlihua experimental animal technology co. Ltd, Beijing, China) were treated with U251 cells (2.0×10^6^) via subcutaneous injection. When the tumor reached 70 mm^3^, all mice were randomized into four groups: 1) Control group, treated with 100 μL of saline; 2) CQ group, treated with 20 mg/kg/day CQ in 100 μL; 3) MOX group, treated with 20 mg/kg/day MOX in 100 μL; 4) MOX+CQ group, treated with 20 mg/kg/day CQ combined with 20 mg/kg/day MOX in 100 μL. All drugs were administered via intraperitoneal injections every day. Tumor size was measured with vernier caliper and calculated as volume (mm^3^) using the equation: V=0.5×length×width^2^. After 24 days, all mice were euthanized with ether anesthesia, and tumors were dissected and frozen in liquid nitrogen.

### Immunohistochemistry

Solid tumors were removed from sacrificed mice and embedded into paraffin (Citotest, China). Paraffin-embedded tumor tissues were sectioned to 5 μm thickness and labeled with antibody LC3B, Ki67 by HRP-conjugated secondary antibody. Immunoreactivity was visualized by incubation with DAB. Hematoxylin was used for background counterstaining. Images were acquired using fluorescence microscopy (Feica, Germany) and analyzed with ImageJ software (version 2.0).

### TUNEL assays

Tumor paraffin-embedded sections were stained with the TUNEL technique using an *In situ* Cell Death Detection Kit to evaluate the apoptotic response of tumor tissues, Fluorescein (Roche Diagnostics, Mannheim, Germany). After being deparaffinized and hydrated, slides were washed with PBS twice and incubated with proteinase K (20 μg/ml) for 25 min at 37°C. After a second round of washes, slides were incubated with TUNEL reaction mixture prepared freshly for 1 h at 37°C in a moist chamber. Images were acquired using fluorescence microscopy (Feica, Germany).

### Quantitative and statistical analysis

For quantification, the intensity of bands in western blot was measured by ImageJ software (version 2.0). The values subsequent to normalizing to the loading control in the control groups were set as 1.0. LC3B, Ki67 staining intensity was measured from the number of positive cell nuclei in 25% fields using ImageJ software.

All experiments were repeated at least three times. Data are presented as the mean ± standard deviation (SD). One-factor analysis of variance (ANOVA) test was used to assess the differences among all the experiment and control or MOX groups. GraphPad Prism Package (version 5.0) and SPSS version 20.0 statistical software were used for statistical analysis. *P*<0.05 was considered to be indicative of a statistically significant difference.

## Results

### MOX stimulated autophagy of glioma cells *in vitro*

**Figure 1:**
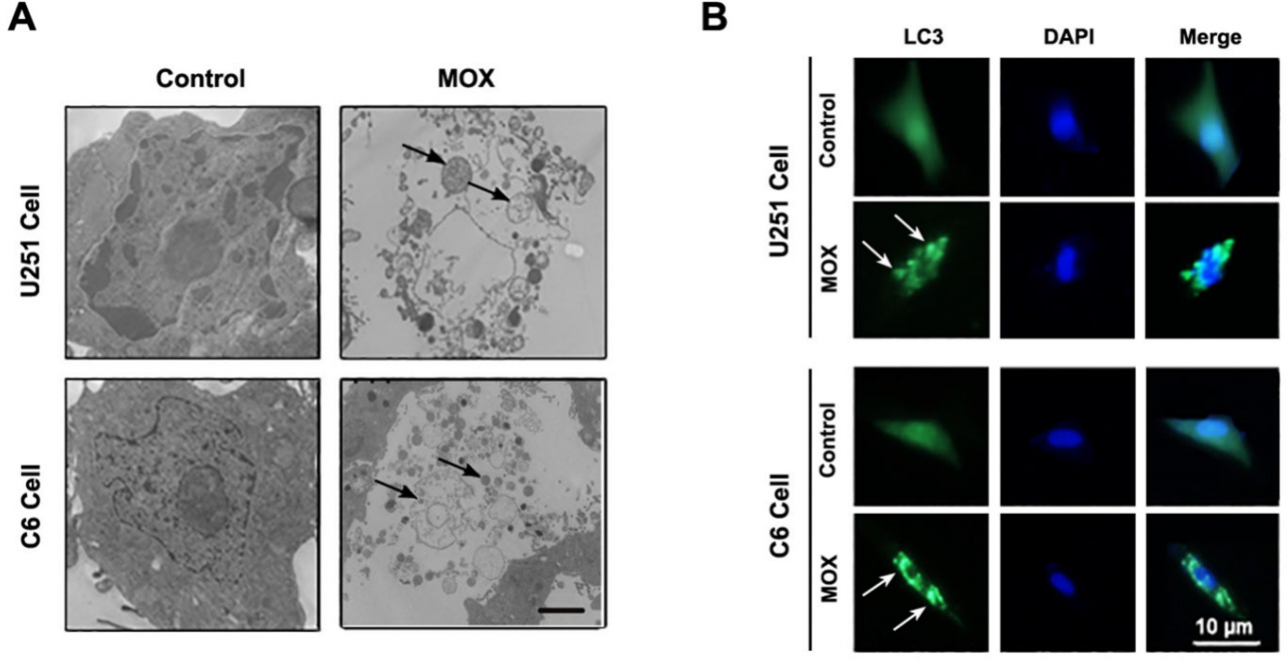
MOX stimulated autophagy of glioma cells *in vitro*. (**A**) U251 and C6 cells were treated with MOX (0, 20 µM) for 48 h and analyzed by TEM. (**B**) U251 and C6 cells were transfected with GFP-LC3 plasmid then the cells were treated with DMSO (< 0.1%) or 20 µM MOX for 48 h. To determine the autophagic response, cells were inspected at 40× magnification for numbers of GFP-LC3 puncta. Scale bar, 10 µm.

The effect of MOX on the induction cell autophagy in glioma cells was examined by TEM and GFP-LC3 transfection. As shown in Fig. 1A, TEM revealed obviously characteristic autophagosomes in U251 and C6 cells treated with MOX but not in control cells. Arrows indicated autophagosomes containing intact and degraded cellular debris. What's more, we determined the induction of autophagy by GFP-LC3 transfection. As shown in Fig. 1B, we observed abundant LC3 puncta in 48 h MOX-treated cells compared with untreated U251 and C6 cells. These data demonstrated that MOX stimulated autophagy of glioma cells *in vitro*.

### Dose-dependent effect of MOX on an autophagy-related protein LC3

**Figure 2:**
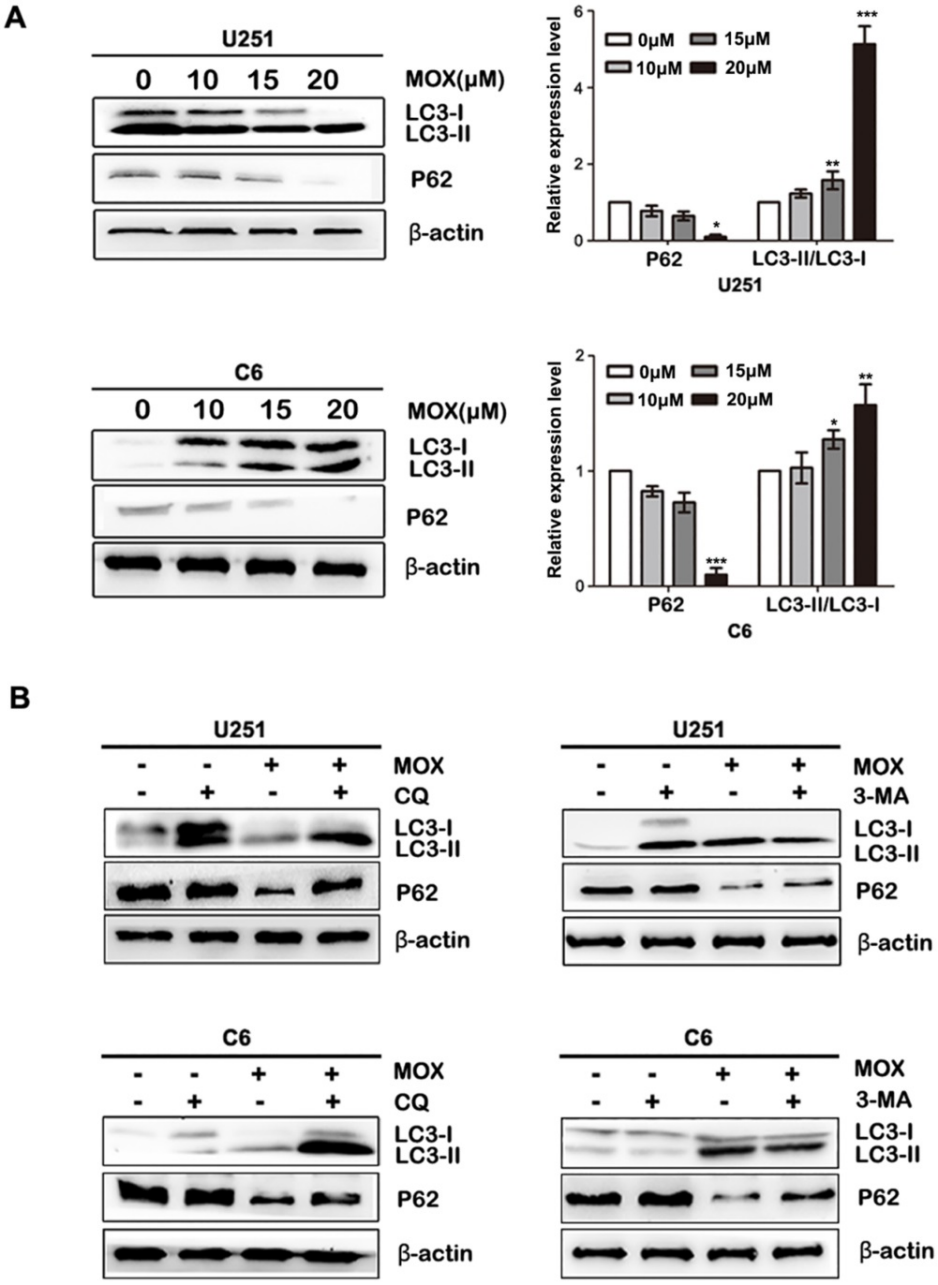
Dose-dependent effect of MOX on an autophagy-related protein LC3 (**A**) U251 and C6 cells were exposed to different concentrations (0, 10, 15, 20 µM) of MOX for 48 h. The LC3B and P62 levels were examined by western blot. (**B**) U251 and C6 cells were treated with 20 µM MOX in the absence or presence of CQ (15 µM) or 3-MA (5 µM) for 48 h. LC3B and P62 expressions were examined by western blot. Data were presented as the means ± SD of three independent tests. *P < 0.05, **P < 0.01, ***P < 0.001 as compared with control group. β-actin was used as an internal standard.

In order to investigate the mechanism involved in MOX-mediated cell autophagy, the proteins related to cell autophagy were measured by western blot. As shown in Fig. 2A, LC3-II/LC3-I expression was more pronounced, and the level of P62 was decreased in U251 and C6 cells with an increased dose of MOX. Next, to explore whether MOX-induced of LC3 LC3-II/LC3-I is due to autophagy induction or the inhibition of autolysosomal function, CQ and 3-MA were used to inhibit autophagic flux. As shown in Fig. 2B, pretreatment with 3-MA resulted in the decrease of LC3-II/LC3-I compared with the MOX group in U251 and C6 cells. Meanwhile, inhibition of autophagy by using CQ, enhanced the expression of LC3-II/LC3-I compared with MOX use alone. Taken together, the result suggested that MOX could stimulate autophagy of U251 and C6 cells in a dose-dependent manner.

### AKT/mTOR pathway is involved in MOX-induced autophagy of glioma cells

**Figure 3:**
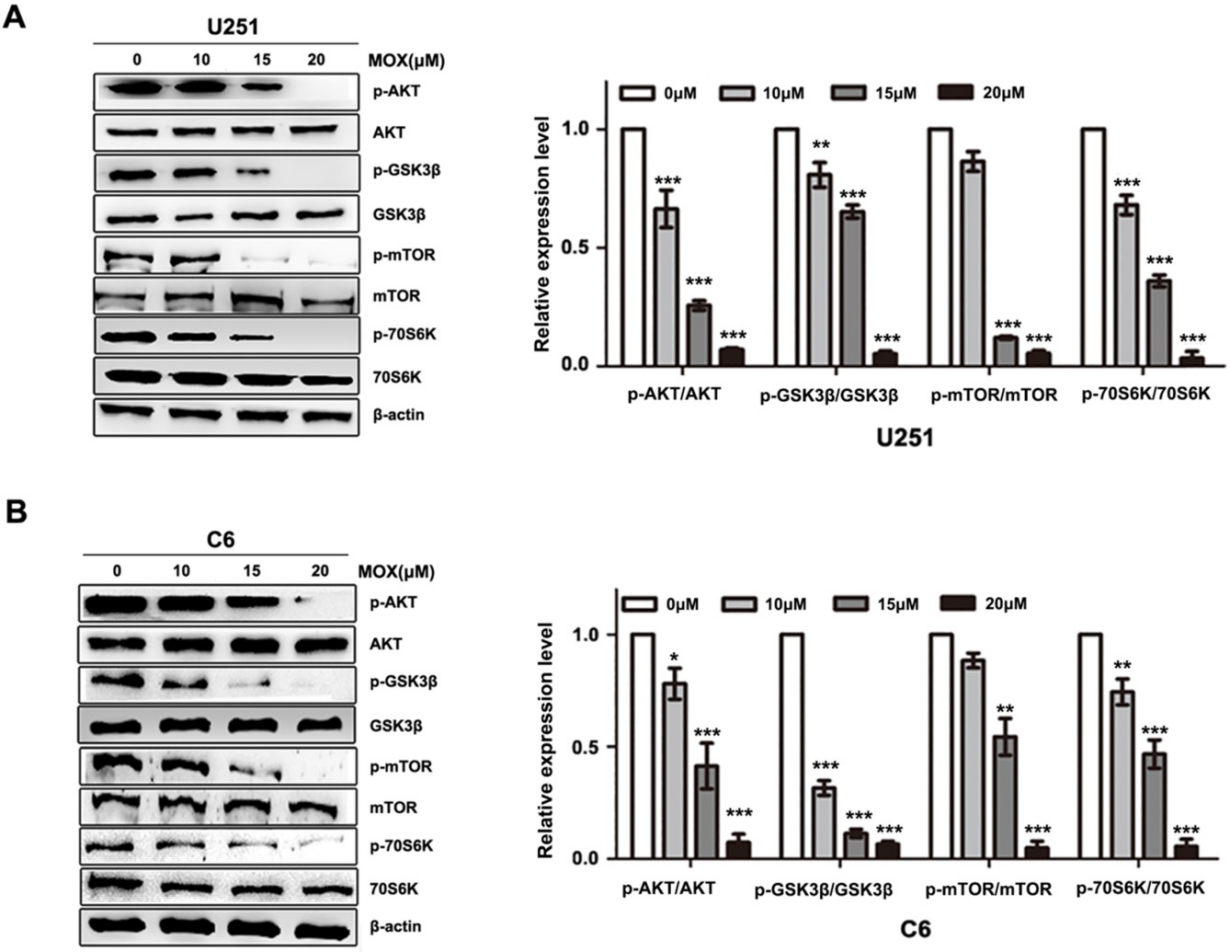
AKT/mTOR pathway is involved in MOX-induced autophagy of glioma cells. (**A, B**) U251 and C6 cells were incubated with MOX at different concentrations (0, 10, 15, 20 µM) for 48 h, the effects of MOX on the levels of AKT, p-AKT (Ser473), mTOR, p-mTOR (S2448), P70S6K, p-P70S6K (Ser417), GSK3β, p-GSK3β were examined by western blot. Data were presented as the means ± SD of three independent tests. *P < 0.05, **P < 0.01, ***P < 0.001 as compared with control group. β-actin was used as an internal standard.

To elucidate the underlying mechanism of MOX on autophagy, AKT/mTOR pathway was investigated in MOX-induced autophagy. Compared with control group, p-AKT (Ser473) and p-mTOR (S2448) were decreased with MOX treatment in a dose-dependent manner, and the downstream protein p-p70S6K (Ser417) and p-GSK3β were reduced in U251 and C6 cells (Fig. 3A, B). These data together implied that AKT/mTOR pathway is involved in MOX-induced autophagy in U251 and C6 cells.

### Inhibition autophagy repressed MOX-induced apoptosis in glioma cells

**Figure 4:**
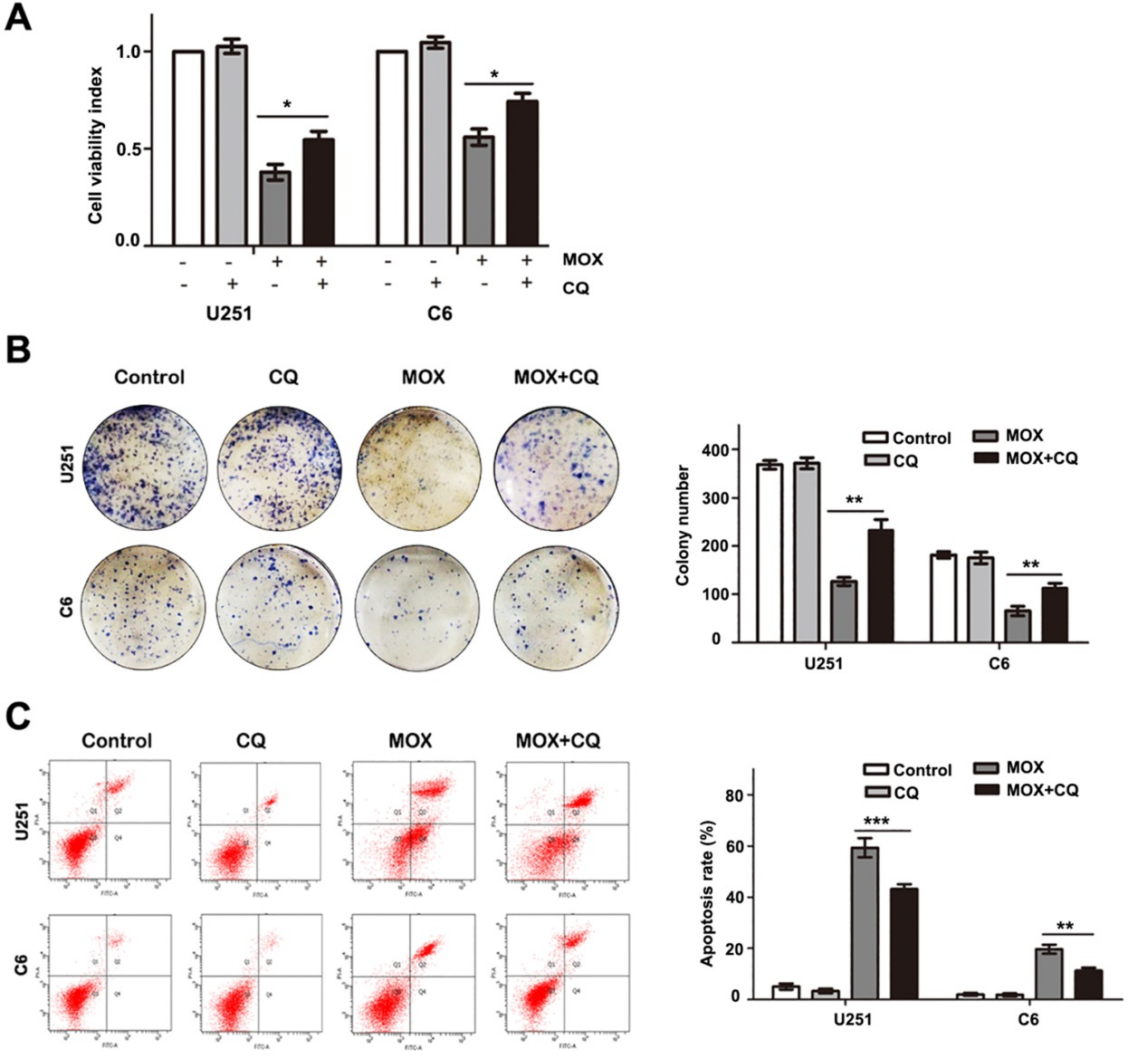
Inhibition of autophagy repressed the apoptosis effect of MOX on glioma cells. U251 cells and C6 cells were pretreated with CQ (15 µM) for 1 h before being exposed to 20 µM MOX. Cell viability was measured by MTT (**A**) and colony formation analysis (**B**). (**C**) The percentage of apoptotic cell was evaluated by flow cytometry after cells were incubated with MOX (20 µM) in the presence or absence of CQ (15 µM). Q2 plus Q4 areas were calculated as the apoptosis ratio. Data were presented as the means ± SD of three independent tests. **P < 0.01, ***P < 0.001 as compared with MOX group.

To determine the role of autophagy in MOX-induced cell apoptosis, we treated cells with CQ for 1 h to block autophagy before MOX treatment. Cell growth was assessed by MTT assay and colony formation analysis. As shown in Fig. 4A-B, co-treatment with CQ, the cell growth ability was increased in U251 and C6 cells. Then, flow cytometry analysis was performed using Annexin V-PI double staining. As shown in Fig. 4C, inhibition of autophagy by CQ in MOX-treated cells reduced the percentage of apoptotic cells compared with those treated with MOX alone. Specifically, the fraction of apoptotic cells reduced from 59.10±5.31% in MOX-treated U251 cells to 45.50±2.65% in co-treated cells and the apoptotic cell percentage reduced from 17.02±2.49% to 11.70%±1.67 in C6 cells. These results showed that inhibition of autophagy repressed MOX-induced apoptosis in U251 and C6 cells.

### MOX induced autophagy *in vivo*

**Figure 5:**
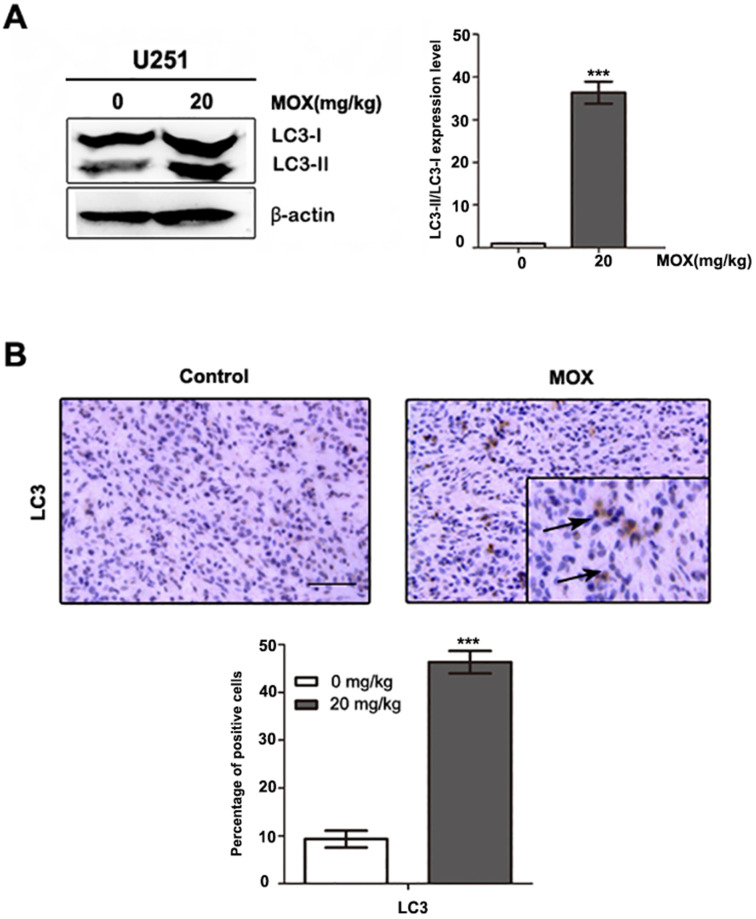
MOX induced autophagy of glioma cell *in vivo*. (**A**) Orthotopic xenograft tissues were extracted to assess the levels of LC3-II by Western blot analysis. (**B**) LC3 expression in orthotopic xenografts was examined by immunohistochemical. Arrows indicate positive cells which stained brown. Scale bar, 50 µm. Data were presented as the means ± SD of three independent tests. β-actin was used as an internal standard. ***P < 0.001.

A series of therapeutic experiments were conducted in U251 cell xenograft mouse models. As shown in Fig. 5A, a massive LC3B accumulation was detected on tumor sections in MOX-treated xenografts compared with the control group. Meanwhile, we observed LC3-II expression was more pronounced in MOX-treated tumors (Fig. 5B). Together, these findings showed that MOX obviously induced autophagy *in vivo*.

### Inhibition autophagy reduced the apoptosis effect of MOX *in vivo*

**Figure 6:**
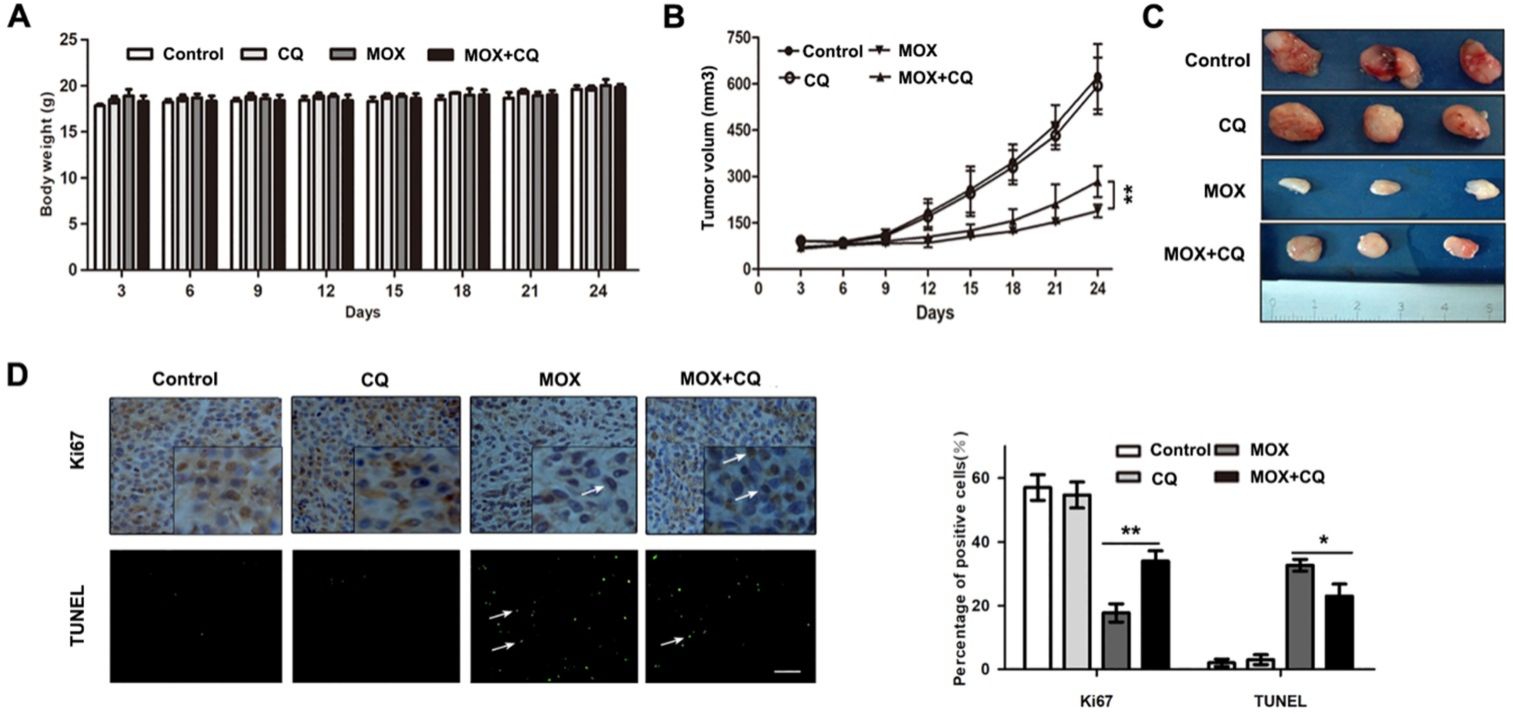
Inhibition of autophagy reduced the apoptosis effect of MOX on glima cell *in vivo*. (**A**) Charting of mouse weight with time. Mice were sacrificed days after the indicated treatments, the tumor volume (**B, C**) were measured. (**D**) Immunohistochemistry staining result of Ki67 and TUNEL assay on tumor sections. Scale bar, 50 µm. Data were presented as the means ± SD of three independent tests. *P < 0.05, **P < 0.01.

Next, to confirm the potential effect of autophagy induced by MOX on glioma tumor *in vivo*, an internal engraftment model was established and tested. As shown in Fig. 6A, no significant difference in the weights of the mice was observed between the experimental groups after all measured days. As shown in Fig. 6B/C, there is no significant difference in the tumor weight or volume between the control and CQ groups, but tumor weight and volume in MOX group were markedly lower than in control group. Furthermore, compared with MOX group, co-treatment of MOX and CQ was less effective in reducing the tumor size. Finally, as shown in Fig. 6D, TUNEL assay demonstrated a greater number of dead cells and an evident increase in the apoptotic proportion in MOX treated tumor tissues compared with the MOX+CQ treated tumor tissues. Immunohistochemistry was performed to label Ki 67, which was used to confirm the change in the proliferation status of the tumors. MOX+CQ xenografts displayed stronger Ki67 staining compared with that of MOX treated mice. The result demonstrated the less dead cells in MOX+CQ group compared with the MOX group. In generally, these data recapitulated the observations made *in vitro* and showed that autophagy increased the apoptotic effects of MOX on U251 *in vivo*.

## Discussion

Gliomas are one of the primary brain tumors 27. In our previous work, we proved that MOX could induce apoptotic death in glioma cells, MOX may represent a potent and promising agent to combat glioma 13. Recent researches have demonstrated that autophagy can improve anti-cancer therapy and have an integral role in tumor maintenance. Research into autophagy is underway to better direct therapies 28, 29. In this study, we first described autophagy phenomenon in glioma cells after MOX treatment. Second, we explored the mechanisms involved in MOX-mediated cell autophagy. Third, the relationship between cell apoptosis and autophagy in glioma cells treated with MOX was discussed.

Autophagy is a key mechanism for maintaining cell homeostasis by adjusting cell metabolism to nutrient supply and removing damaged organelles 30. Research has demonstrated that chemotherapy or radiotherapy could activate autophagy 31. For example, Liu R et al. showed that itraconazole inducted autophagy in glioblastoma cells 32, and Zhao WY et al. demonstrated that isogambogenic acid inhibited the growth of glioma through activating autophagy 33. Conversion of LC3-I into LC3-II is widely used as a marker for autophagosome formation. LC3-I is the soluble form while LC3-II is the autophagosome membrane bound form 34. P62 is incorporated in completed autophagosomes and degraded in autolysosomes 35. In this study, we found MOX could induce autophagy in U251 and C6 cells by TEM, GFP-LC3 transient transfection and western blot analysis. In addition, Li CG et al. proved that 3-MA inhibited the initial step of autophagy, and CQ inhibited autophagosome-lysosome fusion and degradation steps in glioblastoma Cells 36. Therefore, CQ and 3-MA were used as autophagy inhibitors to investigate MOX-induced up regulation of LC3B because of autophagy induction. Thus, we show that MOX induced autophagy in U251 and C6 cells.

Furthermore, we explored the signalling pathways regulating autophagy. The well characterized AKT/mTOR pathway is an attractive therapeutic target that contributes to the initiation and maintenance of cancer 37. AKT signalling has an important role in the regulation of cell proliferation, angiogenesis, migration and invasion. Chronologically, AKT can stimulate p-mTOR that is an upstream protein 38-40. Cell growth is partly regulated through mTOR 41, 42. In our study, p-AKT, p-mTOR, p-p70S6K and p-GSK3β were decreased in U251 and C6 cells after MOX treatment. These results further indicated that MOX induced autophagy in U251 and C6 cells through the AKT/mTOR signalling pathway.

Some studies indicated that autophagy induced by therapeutic interventions can cause the death of cancer cells that are resistant to apoptosis 43-46. However, the relationship between autophagy and apoptosis in cancers is complex. In our previous study, MOX could induce cell apoptosis of glioma cells. Thus, the relationship of apoptosis with autophagy is deemed necessary for investigation. The autophagy flux of glioma was inhibited by CQ which could decrease lysosomal function 47, 48. The results indicated that the effect of autophagy inhibitor involved in autophagy and apoptosis. The inhibition of autophagy increased the cell ability measured by MTT and colony formation assay, and reduced apoptosis measured by flow cytometry in glioma cells. These findings suggested that autophagy played a significant role in MOX-induced cell death and that inhibition autophagy observably reduced the anti-tumor effects of MOX.

At the same time, the mechanisms of MOX in mouse xenograft models were performed. Previous study showed that MOX resulted in inhibition of the tumor growth without overall gross toxicity. The selected dose of the MOX injections was 20 mg/kg, and MOX could reduce the tumor mass of U251 xenografts 13. Western blot and immunohistochemistry analysis confirmed the increase in LC3B following MOX treatment. These results demonstrated that MOX can induce autophagy of glioma *in vivo*. It is reported that CQ can be regarded as autophagy inhibitor *in vivo* 49, 50. In follow-up experiment, we selected the dose of CQ injections as 20 mg/kg, where it did not exert a significant inhibitory effect on tumor growth. In this study, it was found that the intraperitoneal injection of MOX prominently inhibited the growth of glioma cells compared with MOX+CQ group and neither showed any abnormality in behavior nor significant major organ-related toxicity *in vivo*. As a result, we recapitulated the observations made *in vitro* and showed that autophagy enhanced the anti-glioma treatment effects of MOX. Further studies in glioma cancer animal models as well as in human clinical trials are necessary.

In conclusion, our results demonstrated that MOX can induce autophagy in glioma cells while exploring some of the underlying molecular mechanisms. Moreover, we stated that the AKT/mTOR signalling pathway contributed to MOX-induced autophagy in glioma cells. Apoptosis was an important cause of death of glioma cells, while autophagy induced by MOX promoted apoptosis. This study provided a new prospect for the application of MOX and modulating autophagy may represent an effective strategy for the treatment of glioma.
