# Moxidectin induces Cytostatic Autophagic Cell Death of Glioma Cells through inhibiting the AKT/mTOR Signalling Pathway: Erratum

**DOI:** 10.7150/jca.86757

**Published:** 2023-07-06

**Authors:** Jingjing Liu, Hongsheng Liang, Saadia Khilji, Haitao Li, Dandan Song, Chen Chen, Xiaoxing Wang, Yiwei Zhang, Ning Zhao, Xina Li, Aili Gao

**Affiliations:** 1 School of Life Science, Northeast Agricultural University, Harbin, Heilongjiang, China.; 2 Department of Neurosurgery, The First Affiliated Hospital of Harbin Medical University, Harbin, Heilongjiang, China.; 3 Department of Cellular and Molecular Medicine, Faculty of Medicine, University of Ottawa, Ottawa, Ontario, Canada.; 4 Department of Pharmacy, The First Affiliated Hospital of Harbin Medical University, Harbin, Heilongjiang, China.; 5 College of Life and Health Sciences, Northeastern University, Shenyang, Liaoning, China.

Recently, we sorted out all the original data and found that several figures in our article have errors during image assembly. In our paper, we found mistakes in that the duplicate images were inadvertently used in Figure 2B (β-actin) and Figure 3B (AKT) blot. We also found more representative and higher pixel images of colony formation (C6-control; C6-CQ) and flow cytometry (U251-Control; U251-CQ; U251-IVM; U251-IVM+CQ; C6-Control; C6-CQ) images in Figure 4B, C. Meanwhile, we incorrectly used the Ki67 (Control; CQ; MOX; MOX+CQ) and TUNEL (MOX; MOX+CQ) images in Figure 6D.

The correct images are provided below. This correction will not affect the results and conclusions. All the authors of the paper have agreed to this correction. The authors apologize for any inconvenience this may have caused.

## Figures and Tables

**Figure 2:**
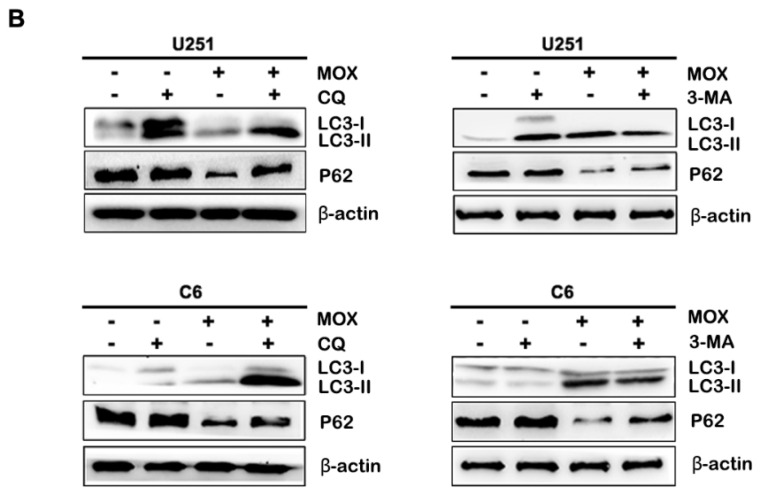
Dose-dependent effect of MOX on an autophagy-related protein LC3. (**B**) U251 and C6 cells were treated with 20 µM MOX in the absence or presence of CQ (15 µM) or 3-MA (5 µM) for 48 h. LC3B and P62 expressions were examined by western blot. Data were presented as the means ± SD of three independent tests. β-actin was used as an internal standard.

**Figure 4:**
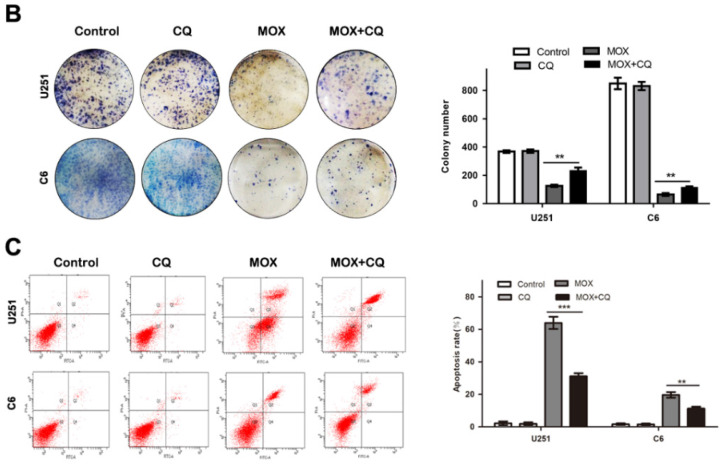
Inhibition of autophagy repressed the apoptosis effect of MOX on glioma cells. Cell viability was measured by colony formation analysis (**B**). (**C**) The percentage of apoptotic cell was evaluated by flow cytometry after cells were incubated with MOX (20 µM) in the presence or absence of CQ (15 µM). Q2 plus Q4 areas were calculated as the apoptosis ratio. Data were presented as the means ± SD of three independent tests. **P < 0.01, ***P < 0.001 as compared with MOX group.

**Figure 6:**
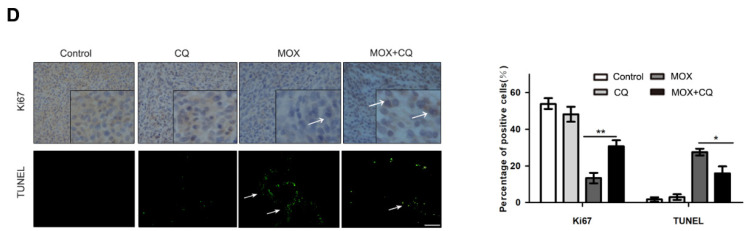
Inhibition of autophagy reduced the apoptosis effect of MOX on glioma cell* in vivo*. (**D**) Immunohistochemistry staining result of Ki67 and TUNEL assay on tumor sections. Scale bar, 50 µm. Data were presented as the means ± SD of three independent tests. *P < 0.05, **P < 0.01.

